# Optimal control of complex atomic quantum systems

**DOI:** 10.1038/srep34187

**Published:** 2016-10-11

**Authors:** S. van Frank, M. Bonneau, J. Schmiedmayer, S. Hild, C. Gross, M. Cheneau, I. Bloch, T. Pichler, A. Negretti, T. Calarco, S. Montangero

**Affiliations:** 1 Vienna Center for Quantum Science and Technology, Atominstitut, TU Wien, Stadionallee 2, A-1020 Vienna, Austria; 2 Max-Planck-Institut für Quantenoptik, Hans-Kopfermann-Str. 1, 85748 Garching, Germany; 3 Laboratoire Charles Fabry, Institut d’Optique - CNRS - Université Paris Saclay, 91127 Palaiseau, France; 4 Institute for complex quantum systems & Center for Integrated Quantum Science and Technology (IQST), Universität Ulm, Albert-Einstein-Allee 11, D-89075 Ulm, Germany; 5 Zentrum für Optische Quantentechnologien and The Hamburg Centre for Ultrafast Imaging, Universität Hamburg, Luruper Chaussee 149, D-22761 Hamburg, Germany

## Abstract

Quantum technologies will ultimately require manipulating many-body quantum systems with high precision. Cold atom experiments represent a stepping stone in that direction: a high degree of control has been achieved on systems of increasing complexity. However, this control is still sub-optimal. In many scenarios, achieving a fast transformation is crucial to fight against decoherence and imperfection effects. Optimal control theory is believed to be the ideal candidate to bridge the gap between early stage proof-of-principle demonstrations and experimental protocols suitable for practical applications. Indeed, it can engineer protocols at the quantum speed limit – the fastest achievable timescale of the transformation. Here, we demonstrate such potential by computing theoretically and verifying experimentally the optimal transformations in two very different interacting systems: the coherent manipulation of motional states of an atomic Bose-Einstein condensate and the crossing of a quantum phase transition in small systems of cold atoms in optical lattices. We also show that such processes are robust with respect to perturbations, including temperature and atom number fluctuations.

The last two decades have seen exceptional progress in the ability to engineer, manipulate and probe complex quantum systems. The concepts of quantum computation, quantum simulation or precision measurement beyond the classical limit have been validated in the laboratory and quantum sensing and metrological devices have been developed for specific applications^1^,^2^,^3^,^4^,^5^,^6^. In order to fully exploit the potential of complex quantum systems, designing more robust and efficient experimental protocols is an important challenge.

Most protocols developed so far in research laboratories rely on analytic or simple empirical solutions and are far from being optimal. For instance, slow transformations are highly sensitive to decoherence and experimental imperfections. It would therefore be desirable to have at our disposal a method to design fast and arbitrary complex manipulations. In addition to this, to be experimentally sound, such a method would have to be robust with respect to system perturbations. This challenge can be met by means of optimal control theory, that is, the automated search of an optimal control field to steer the system towards the desired goal^7^,^8^. Quantum Optimal Control (QOC) has been applied very successfully in the case of few-body quantum systems: it has been shown that QOC can steer the dynamics in the minimum allowed time and that the optimal protocols are robust with respect to noise^8^. In particular, it has been experimentally demonstrated, for quantum dynamics taking place in an effective two-level system, that QOC allows to saturate the Quantum Speed Limit (QSL) – the minimal time necessary to transform one state into another for a given energy of the driving^9^,^10^,^11^,^12^,^13^,^14^. However, only recently QOC has been extended to embrace many-body quantum dynamics in non-integrable quantum systems^15^,^16^,^17^,^18^,^19^; the feasibility of an optimal transformation at the QSL in many-body quantum systems has never been experimentally verified. Enabling such a step is of major importance to develop optimal quantum protocols for systems consisting of many elements. Since few-body quantum systems can always be efficiently simulated classically, protocols for large quantum systems are needed to demonstrate the so-called ‘quantum supremacy’ – a quantum technology which outperforms the corresponding classical one.

In this paper, we apply QOC to two ultracold atom systems undergoing complex transitions in the non-perturbative regime (as depicted in Fig. 1) and we show that it is possible to speed up their dynamics at timescales comparable with their QSL theoretical estimates. The two selected experiments are prime examples of quantum systems in which the dynamics is affected by interactions between particles and where much is to be gained from speeding up the desired transformation. Given the diversity of the two physical systems, of the dynamical processes, and also of the theoretical models used to describe them, our results support the vision that QOC will become a general approach to engineer quantum protocols at the fundamental and eventually at technological level.

In the first experiment, we demonstrate a fast control scheme for the motional state of a quasi-one dimensional Bose–Einstein condensate (BEC) on an atom chip (Fig. 1b). In this experiment, and in general in the common case of driving a transition between two energy levels of a system, an intuitive protocol (for example an adiabatic solution) does not necessarily exist. The transition can, under certain constraints, be driven by a Rabi pulse at the frequency of the level splitting. However, in the presence of other accessible levels or loss processes, this option has a strongly limited efficiency. Indeed, the complexity of the first experiment we present arises from the multiplicity of accessible motional states and the non-linearities induced by atom-atom interactions. We show that, by using a mean-field Gross-Pitaevskii description of the system, QOC is successful at optimizing state transformations or state preparation, making it a versatile tool for potential quantum information processing applications. Following an optimized non-trivial trajectory, we achieve theoretical and experimental infidelities below 1%. Let us note that the optimized transfer reported here corresponds to the initialization step of a twin-atom beams source. This transfer is done in 1.09 ms, which is a major improvement compared to the 5 ms previously needed^20^,^21^, since it allows separating the timescales of source initialization and twin beams emission.

In the second experiment, we speed up the crossing of the finite-size one-dimensional superfluid Mott-insulator (SF-MI) crossover of cold atoms in an optical lattice (Fig. 1c). In this scenario, and in general in any crossing of a quantum phase transition, i.e. any adiabatic quantum computation scheme, adiabatic manipulations are generally applied. Although maintaining adiabaticity is impossible in the thermodynamic limit, almost adiabatic transformations can become feasible for finite size systems. However, they are – by definition – slow compared to the typical timescales of the system. Speeding up the transformation can lead to a significant gain in that regard. We simulate and optimize the dynamics by means of time-dependent Density Matrix Renormalization Group simulations and demonstrate the power of QOC to efficiently treat many-body dynamics: we cross the phase transition on a time scale compatible with the QSL — about one order of magnitude faster than the adiabatic protocol — while maintaining the same final state fidelity. This experiment is the first example of QOC applied to the crossing between different phases at the QSL and might have implications to improve the efficiency of future adiabatic quantum computation protocols.

As we will show, in both experiments the optimal transformations we engineer are robust with respect to finite temperature and moderate fluctuations of the systems’ parameters, such as the atom number, a fundamental requirement for a successful application of optimal protocols to experimental systems.

## Optimal Control and Quantum Speed Limit

**Figure 1:**
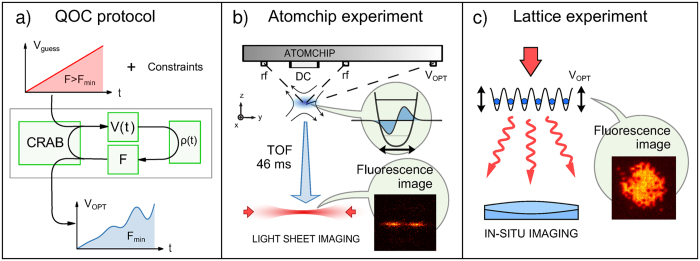
(**a**) The optimal control algorithm (CRAB, see Sec. 1.1) applies a first control field *V*_guess_(*t*) to a numerically simulated experiment. Taking into account experimental constraints, it optimizes the control field *V*(*t*) relying on the figure of merit *F* after time evolution. The final control field obtained after optimization, *V*_OPT_(*t*), optimally steers the system in the minimal possible time *T*_OPT_ compatible with the theoretical and experimental limitations. (**b**) Vienna atomchip experimental setup: illustration of the experimental setup with the atomchip (top) used to trap and manipulate the atomic cloud (middle) and the light sheet as part of the imaging system (bottom). The trapping potential, centered on a DC wire, is made slightly anharmonic by alternating currents in the two radiofrequency (rf) wires. It is then displaced (black arrow) along the optimal control trajectory *V*_OPT_(*t*), using an additional parallel wire located far away from the DC wire and carrying a current proportional to *V*_OPT_(*t*). By this mechanical displacement of the wavefunction, a transition is realized from the ground to the first excited state of the trap. The atomic cloud is imaged after a 46 ms time-of-flight. (**c**) Garching lattice experiment setup: an optical lattice is applied along an array of tubes and drives the superfluid to Mott-insulator transition with one atom per site (top) following the optimized control field *V*_OPT_(*t*) applied to the lattice depth (black arrows). The distribution of atoms in the Mott regime is probed by fluorescence imaging through a high-resolution microscope objective with single-site resolution and single-atom sensitivity (bottom right).

A typical optimal control problem is defined as follows: given a dynamical law which defines the time evolution of the system state *ρ* and depends explicitly on an external control field *V*(*t*), one looks for the optimal control field *V*_OPT_(*t*) such that a quantity of interest — the figure of merit *F*(*V*(*T*)) — is extremized at the final time *T* (Fig. 1a). In the following, among the different algorithms that have been successfully developed to perform QOC processes^8^,^22^, we will exploit an approach recently introduced by some of us^15^,^23^: the Chopped RAndom Basis (CRAB) optimization. This approach has been specifically designed to solve optimal control problems where access to the knowledge of the system properties is limited and/or the computation of the figure of merit is highly demanding (see appendix A for details): for example when using tensor network methods^24^,^25^, multi-configuration time-dependent Hartree Fock methods^26^,^27^,^28^, or in a closed-loop setting whenever the optimization is performed directly as part of the experimental cycle^16^.

Despite the successes of QOC, there are fundamental limitations that clearly cannot be overcome. One of the most fundamental ones is related to the energy-time uncertainty – the Quantum Speed Limit (QSL). It accounts for the fact that the system’s finite energy defines a minimal time-scale needed for the system to react^9^. The simplest instance of such fundamental limit is, in a two-level system undergoing a Rabi oscillation, the Rabi frequency which provides a lower bound for the time needed to perform a transition between the levels. More generally, it can be proven that the time needed to perform a given transformation between two states is bounded by 
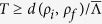
, where *d*(*ρ*_*i*_, *ρ*_*f*_) is the distance between the initial and the final states, and 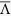 is the time-averaged p-norm of the Liouvillian^9^,^10^,^11^,^12^,^13^. Whenever the previous bound is saturated, the system is said to be driven at the QSL^29^. An independent heuristic estimate of the QSL can be provided by solving the (constrained) optimal control problem at fixed control field strength but for different total transformation times *T*: the minimal time needed to reduce the figure of merit below some critical threshold can be defined as the QSL of the process. It has been shown in a few cases that these two definitions coincide^28^,^29^,^30^. The functional dependence with the transformation time *T* of the figure of merit depends on the specific trajectory followed by the system during its time-evolution. When the time-optimal transformation follows the geodesic in the Hilbert space between the initial and the final (orthogonal) state at maximum constant speed, one can derive a remarkably simple expression: 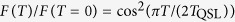 for *T* ≤ *T*_QSL_^28^,^29^,^30^. Finally, in the specific case of a quantum phase transition crossing, an independent estimate of the QSL can be achieved via the minimal gap Δ*E*_*m*_ between ground and first excited states of the system while changing the driving parameter *T*_QSL_ = *πħ*/Δ*E*_*m*_^30^.

**Figure 2:**
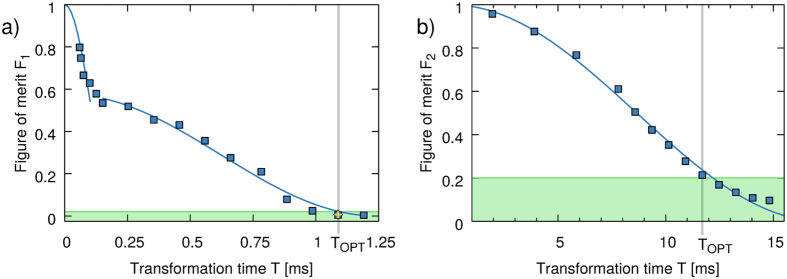
(**a**) Theoretical prediction of the optimal figure of merit *F*_1_ (infidelity with respect to the goal state, see Eq. (2)) achieved by optimal driving of the ground-to-first-excited state transfer of a condensate for *N* = 700 atoms, as a function of the transformation time T (blue squares). The blue solid lines are fits of the numerical results according to 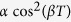 with 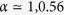 and 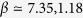 (left and right curve respectively), determining the QSL. The green region represents the smallest measurable infidelity in the experiment. Its intercept with the blue line defines the optimal time *T*_OPT_ = 1.09 ms (gray vertical line), which is the fastest time compatible with both the QSL and the experimental limitations. The yellow cross marks the experimental result performed at *T*_OPT_. (**b**) Theoretical prediction of the optimal figure of merit *F*_2_ (averaged atom number fluctuations in each site at the center of the trap, see Eq. (4)) as a function of the control field duration *T* (blue squares) for the crossing of the SF-MI crossover. The blue solid line is a fit determining the QSL, the crossing point between the numerical result. The green region (estimated experimental limitations) defines the optimal time *T*_OPT_ = 12.0(2) (gray vertical line).

Before entering the technical details of our work, we describe briefly the two major results that we have obtained. The first major result of the present study is to show that the simple cosine-square dependence of the final figure of merit on the total duration of the transformation is a robust feature that holds also for trajectories in complex constrained scenarios. This is illustrated in Fig. 2, where the figure of merit obtained by performing a CRAB optimization for different total transformation times *T* is drawn as a function of *T* for the two experimental protocols that we considered (blue squares in Fig. 2). Aside from the rapid decay observed at short total transformation times in Fig. 2a (for which we provide an interpretation in the next section), the numerical data are accurately described by a cosine-square function, which allows us to unambiguously define the theoretical QSL by means of a simple one-parameter fit.

When it comes to confronting the theoretical prediction with the experimental realization, one should of course take into account the deviations from the idealized theoretical model and the concrete measurement capabilities, which introduce a limited distinguishability between states (in terms of any measurable quantity). Hereafter we define the optimal transformation time *T*_OPT_ < *T*_QSL_ as the shortest transformation time for which the figure of merit reaches the minimum value compatible with the experimental resolution.

The second major result of our work is to show that the predicted value of the figure of merit at the optimal time *T*_OPT_ is indeed reached in the experiments. This could be directly verified in the case of the motional control of a quasi-one dimensional BEC, as illustrated in Fig. 2a where the yellow cross marks the experimentally measured figure of merit corresponding to the optimal transformation of duration *T*_OPT_. In the case of the crossing of the SF-MI transition, the reliability of the theoretical prediction was indirectly verified in the sense that the final entropy in the system after a transformation of duration *T*_OPT_ was experimentally indistinguishable from that obtained with a much longer, adiabatic transformation.

In the next two sections, we describe in details the experimental protocols, their modeling and compare the theoretical predictions for the time-optimal transformation with the experimental measurements. We show in particular that the optimal transformations predicted using the CRAB algorithm are robust against small differences between the modeling and the concrete experimental realization, such as finite temperature and finite atom number fluctuations. The two experimental systems that we have studied, their theoretical modeling and the properties of the optimal processes that we have engineered are very different in nature. This diversity supports the conjecture that the optimal driving at the QSL is possible under general conditions. Moreover, we also expect that it should be possible to achieve similar results on different platforms, e.g. in superconducting circuits and trapped ions.

## Results

### Fast manipulation of the motional state of a BEC on an atom chip

The first system for which we demonstrate time-optimized driving is a one-dimensional (1D) BEC of Rubidium 87 atoms on an atom chip, performed at the Technische Universität Wien. Optimal control is used to perform coherent transfers between eigenstates of the transverse confining potential. Such transfers are tools for probing non-equilibrium quantum dynamics and studying decay processes from excited eigenstates, for example the emission of twin-atom beams^20^. Furthermore, coherent manipulation of non-classical motional states allows performing interferometric sequences with these states^31^, and opens perspectives for quantum information operations. For the useful implementation of such operations, as well as to separate the timescales of state initialization and of decay processes, the duration of the optimal control transfers is key. We characterize here the QSL for a full population transfer from the ground to the first excited state, and implement experimentally the predicted shortest possible transfer which allows keeping high transfer efficiency.

The atom chip used in this experiment consists of micro-fabricated structures on a surface, generating magnetic fields to trap neutral atoms^32^. They can produce strongly confining potentials and allow for very precise manipulation of the atoms. These capabilities^32^ are here exploited to produce a well-defined transversally anharmonic potential and to accurately displace the trapping potential along the anharmonic direction, following a trajectory designed by QOC. To realize transfers between the BEC motional states, the anharmonicity is necessary as it lifts the degeneracy between the levels and allows transfers to specific eigenstates of the trap or superpositions thereof. It also induces a coupling between center-of-mass motion and intrinsic motion of the BEC^33^. There is no trivial way to perform these transfers fast, due to the presence of interactions and higher energy levels. In order to constrain the dynamics into the two-level system formed by the initial and target states, the minimum duration for a Rabi driving field — a weak amplitude, sinusoidal displacement at the level spacing frequency — is defined by the detuning between the level spacings, which is on the order of 0.16 kHz. This simple picture is complicated by the interactions, which shift the energy levels and are also responsible for population transfers between eigenstates. The required driving time for a sinusoidal displacement, obtained from numerical simulations, exceeds 9 ms. This is much longer than the timescale of interaction-induced decay into twin-atom beams, about 3 ms for our typical atom number^20^. Therefore, although the initial and final states can be described in a two-level model, the time-optimal transfer trajectory is expected to involve higher motional states. Designing the necessary complex driving fields thus requires the use of QOC.

As long as the decay processes can be neglected the different axes of the potential can be treated as independent, and the steering dynamics can be described by considering only the transverse *y*-direction along which the potential is displaced. Thus, in a mean-field approach, the dynamics of the condensate can be described by an effective 1D Gross–Pitaevskii equation (GPE):


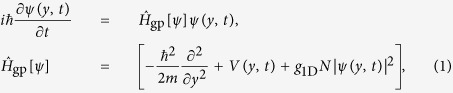


with *N* being the number of atoms in the quasi-condensate and *g*_1D_ = *g*_1D_(*N*) an effective 1D coupling constant for the displacement direction *y* (see appendix B)^34^. The potential along *y* can be well approximated by *V*(*y, t*) = *p*_2_[*y* − *λ*(*t*)]^2^ + *p*_4_[*y* − *λ*(*t*)]^4^ + *p*_6_[*y* − *λ*(*t*)]^6^, where *λ*(*t*) is the control field and the parameters *p*_2_, *p*_4_, *p*_6_ define the trap shape (see appendix C).

The system is initially prepared in the ground state *ϕ*_0_(*y*) of the trap *V*(*y*, 0) with *N* interacting bosons, and the target state is chosen as the corresponding first excited state *ϕ*_1_(*y*). The relevant figure of merit is the infidelity


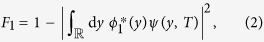


where *ψ (y, T*) is the final state of the system.

We perform a CRAB optimization including the limited bandwidth of the electronics and the maximum possible trap displacement *λ*_max_ = 1 μm. The results for different transformation times *T* are reported in Fig. 2a. The figure of merit *F*_1_ decays abruptly until about 0.15 ms, then more slowly down to its final value at 1.2 ms. This crossover between two characteristic timescales can be understood as follows. For short pulse durations, the infidelity is minimized mainly by displacing the wavefunction and adjusting its phase profile to the one of the target state. This can be done quickly, but is limited to high values of the infidelity. For longer pulse durations, the algorithm can modify the shape of the wavefunction to approach the one of the final state. This process is more time-consuming, but finally yields much better results for the infidelity. The important difference between the timescales of the two transformations (factor ten) leads to the marked inflexion of the figure of merit as a function of the transformation time *T*. We interpret the inflexion point at 0.15 ms as a limit between two optimal transitions, one attempting to optimize through a transformation of phase profile and a displacement of the wave function, the other combining the optimized phase profile to an optimized density profile. Both parts of the curve are compatible with the typical 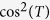 expected from processes at the QSL.

**Figure 3:**
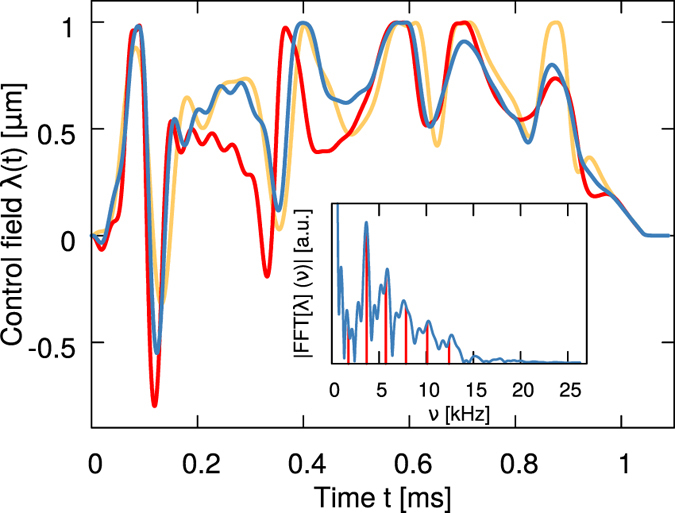
Optimal control fields for transformation times *T* = 1.09 ms for *N* = 1,700 and 7000 atoms obtained via full CRAB optimization (respectively red, blue, and yellow line). Inset: Fourier spectrum of the optimal control field for *T* = 1.09 ms and *N* = 700 (blue solid line). The vertical lines correspond to single particle transitions from the ground state (red).

In summary, the optimal process taking into account experimental constraints and finite measurement precision (on the order of 1%, indicated by the green interval in Fig. 2a) lasts 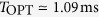 and reaches an infidelity 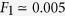. This time-optimal transfer is shorter than previously achieved with approaches using gradient based optimal control techniques^20^,^21^,^35^. It provides a speed-up of about a factor five with respect to the transfer used for previous experiments^20^,^21^. The corresponding optimal control field for *N* = 700 atoms is shown in Fig. 3 (blue line). This control field was used as an initial guess for optimizing two other pulses, for different atom numbers *N* = 1 and 7000. As shown in Fig. 3, the resulting pulses have similar shape but with clear deviations.

To investigate the composition of the optimal transfer control field and gain some physical insight into it, we performed a Fourier analysis of the optimal control field for *T*_OPT_ and *N* = 700 atoms, shown in the inset of Fig. 3. Frequencies beyond ~25 kHz do not play any relevant role. For lower frequencies, the spectrum has a rather continuous behavior with some prominent peaks. We compared the structure of the spectrum with the transitions of the single particle Hamiltonian (vertical lines in the inset). It appears that the main peaks are close to single-particle transitions from ground state to excited states, showing that many eigenstates of the potential are involved in the transfer dynamics. However, not all peaks could be clearly matched with a single physical transition, either single particle or a collective Bogoliubov excitation, see Eq. (5) in appendix B (not shown in the inset of Fig. 3). This analysis emphasizes the complexity of the optimized control field and also the difficulty of engineering and understanding these optimized control fields in intuitive ways, other than the result of an optimization in a complex landscape.

The Fourier analysis of the optimal pulse provides also important information on the information time limit, another fundamental limit of our ability to control quantum systems: it has been shown that the minimal time needed to control a quantum system is bounded from below by the ratio between the effective Hilbert space size *D* and the control bandwidth ΔΩ, such that *T* ≥ *D*/ΔΩ^18^. This information-time limit is in general independent from the QSL and it is hard to estimate in practice. However, we can provide an estimate which shows that the optimal process engineered here is compatible with it. Indeed, the bandwidth of the optimal field, as estimated from Fig. 3, is about ΔΩ~20 *kHz*. The estimate of the relevant Hilbert space size requires more attention. To estimate it, one can project the instantaneous wavefunction onto the bases of the Harmonic trap at time *t* = 0 and get an upper bound of *D*~40. A trivial lower bound is *D*~1, which finally gives 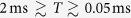, compatible with our findings.

**Figure 4:**
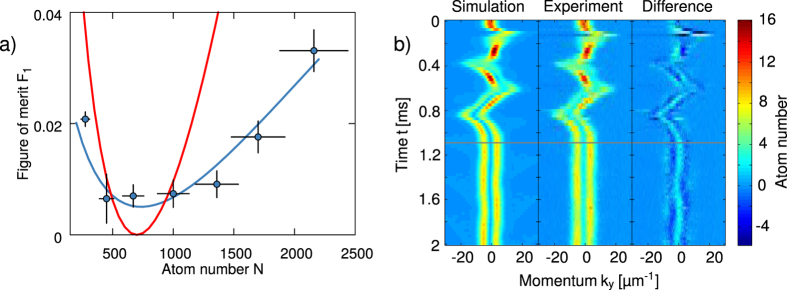
(**a**) Theoretical predictions for the final infidelity *F*_1_ as a function of the atom number when using the control fields optimized for 700 atoms. The shown numerical results are obtained for total transformation times *T* = 1.09, 5.01 ms (blue and red lines) and are compared to experimental results (circles) obtained with the optimal control field for *T*_OPT_ = 1.09 ms. (**b**) Transverse distribution after time-of-flight during the optimal process (*t* < *T*_OPT_) and after (*T*_OPT_ < *t* < 2 ms) for *N* = 700: the experiment (center) and the corresponding GPE simulation (left), plus the residual difference between experiment and simulation (right, only plot with negative values). The gray horizontal line highlights *T*_OPT_.

The optimal control field obtained above, although promising, would be useless if it were not stable against experimental fluctuations. In the present experiment, atom number fluctuations are the main source of perturbation. In normal conditions, fluctuations of the order of 10–20% of the atom number are unavoidable. Therefore, we checked the stability of the optimal process described above against fluctuations of the number of atoms *N*. The numerical results are reported in Fig. 4a, for different atom numbers and different transformation times *T*, one corresponding to the optimal time *T*_OPT_ and another one about five times as long, comparable with the transformation time used in the experiment of ref. 20. As it can clearly be seen, the slower process results in a better theoretical infidelity *F*_1_ at *N* = 700; however its sensitivity against atom number fluctuations is much higher, due to the fact that the effects of atom interactions are integrated over a longer time. Eventually, for atom number fluctuations above 20%, the performances of the slower optimal protocols become even worse than those of the faster ones. In order to further investigate the effects of atom interactions, we also simulated the application of the optimal control field computed for *N* = 1 to the interacting system. We obtained for the optimal time an infidelity *F*_1_ = 0.07, an effect that becomes much worse for the long pulse, with *F*_1_ = 0.25. This result reflects again the fact that the effects of interactions accumulate with time, but also that interactions must be taken into account to obtain good results, including at the optimal time.

#### Experiments

We describe here the experimental test of the optimal process engineered theoretically in the previous section. A BEC is prepared in the transverse ground state of an elongated potential. As illustrated in Fig. 1b, the BEC is trapped and manipulated by current-carrying wires on an atom chip. At any point during the manipulation sequence or after it, the atomic ensemble can be released from the trap and imaged when it crosses a light sheet after a time-of-flight^36^ (for details on the experimental setup and sequence, see appendix C). The profiles of the transverse atomic distribution after time-of-flight are then stacked to construct an image of the time evolution of the transverse momentum distribution during the transfer to the first excited state (Fig. 4b, middle panel). Figure 4b shows a typical experimental result and comparison to simulations: the image in the middle represents the experimental transverse momentum distribution (fluorescence measurement results) as it evolves in time, starting from the beginning of the transfer field. This can be qualitatively compared with the GPE numerical simulation (left panel), or more quantitatively by plotting their difference (right panel). The transfer efficiency is inferred from the evolution of the momentum distribution after the application of the control field, e.g. after *T*_OPT_ = 1.09 ms. This distribution is fitted with Gross-Pitaevskii equation simulations, where the fit parameter of interest is the population in the first excited state. Finally, this analysis yields a transfer efficiency of 99.3(6)%, corresponding to an estimated figure of merit *F*_1_ = 0.7% ± 0.6%, in excellent agreement with the theoretical prediction. We also repeated the experiment with the same control field for different atom numbers, obtaining a good agreement between theory and experiment over one order of magnitude of different atom numbers *N*, as shown in Fig. 4a.

We therefore demonstrated that applying QOC to the atom chip system provides a fast, robust, and efficient method for state initialization and manipulation. This fast state manipulation allowed performing a Ramsey interferometer with motional states^31^. In the next section, we apply the same optimization algorithm to a different system, showing that the optimal steering demonstrated here is not dependent on this particular experimental setup or process, but it shall be expected in general.

### Mott-insulating ground state of bosonic atoms in an optical lattice

The phase transition between the superfluid (SF) and the Mott-insulating (MI) phase in cold atoms has been studied in different experiments^6^, and nowadays the MI state with typically unit filling is used, for instance, as a well-defined initial state for experiments simulating the dynamics of effective spin systems^37^,^38^,^39^. These experiments start with a BEC and cross the phase transition to the desired MI state by slowly increasing the depth of the optical lattice. In an infinite-size homogeneous system these two states are separated by a quantum critical point and therefore cannot be adiabatically connected by varying the lattice depth. In typical experimental systems, however, the finite number of atoms and the presence of an additional confining potential turn the phase transition into a crossover, thereby opening the possibility for an adiabatic preparation of the MI ground state. Here we demonstrate that a faster, non-adiabatic optimal steering across the 1D SF-MI crossover, is possible. The optimized control field we engineer speeds up most of the ramp of the system by a factor of ten compared to the adiabatic protocol, from 12012, without measurable additional distortion of the final state.

The system we use is composed of parallel tubes containing on average 16 Rubidium 87 atoms each. An optical lattice of depth *V*(*t*) is applied along these tubes. Each tube is described by the one-dimensional bosonic Hubbard Hamiltonian^40^


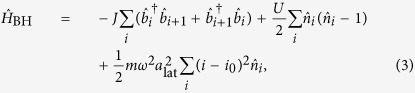


where the index *i* = 1, …, *L* labels the lattice sites and *i*_0_ = (*L* − 1)/2 the center of the trap, *J* is the tunnel coupling between neighboring sites, *U* is the on-site interaction strength, *ω* denotes the harmonic confining potential, and *a*_lat_ the lattice spacing. The operator 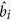 (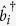) annihilates (creates) an atom at site *i* while 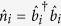 counts the number of atoms at that site. In the absence of the harmonic confinement (*ω* = 0), the critical point of the SF-MI transition is located at *U*/*J* ≈ 3.4^41^, corresponding to a lattice depth *V*_c_ = 4.5 *E*_r_, where 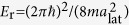 is the recoil energy of the lattice and *m* the atomic mass of the atoms. All the presented theoretical results are obtained in the presence of a trapping frequency *ω* = 2*π* × 63.5 Hz equal to the frequency measured in the experimental setup, and unless stated otherwise, simulating *N* = 16 atoms in a lattice of *L* = 32 sites.

The dynamics of the Hamiltonian (3) is simulated via the time-dependent density matrix renormalization group algorithm (t-DMRG, see appendix D). The time-dependent tunnel coupling *J*(*t*) and the on-site interaction energy *U*(*t*) are derived from the lattice depth *V*(*t*) by calculating the overlap integrals of the Wannier functions for the single-particle problem^40^. The bosonic Hubbard Hamiltonian, provided by Eq. (3), is only a good description of the system for sufficiently large lattice depths (typically *V* > 3 *E*_r_). We therefore assume the system to be initialized at a lattice depth of 3 *E*_r_ and we optimize the functional dependence in time of the lattice depth for a ramp ranging from 3 *E*_r_ to 14 *E*_r_ driving the SF-MI crossover.

The ramp of the lattice depth *V*(*t*) is optimized for different transformation times *T* using the CRAB algorithm. The figure of merit we minimize is the rescaled average variance of the site occupancy in the center of the trap where we expect that the effect of the harmonic potential will be negligible and we could observe a MI state. That is, the figure of merit is defined as


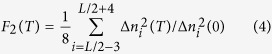


where 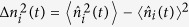 and the sum runs over the eight sites at the center of the harmonic trap. The numerical figure of merit ranges from *F*_2_(*T* = 0) = 1 to *F*_2_ = 0 for a perfect Mott-insulator, while any residual excitations at the final time will increase *F*_2_(*T*). Figure 2b displays the resulting optimal figure of merit *F*_2_ as a function of the transformation time *T*. As expected for an optimal crossing of a quantum phase transition^29^, the numerical results are accurately described by the curve 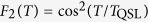, resulting in *T*_QSL_ = 17.3(2) ms. However, due to experimental limitations arising from the non zero temperature of the 1D tubes (green region in Fig. 2b) the optimal verifiable figure of merit corresponds to an optimal time *T*_OPT_~12 ms. Finally, the QSL we have found is compatible with the independent theoretical prediction based on the minimal gap Δ*E*_*m*_: we computed it numerically for a system of *N* = 16 particles *T* = *πħ*/Δ*E*_*m*_~12 ms.

**Figure 5:**
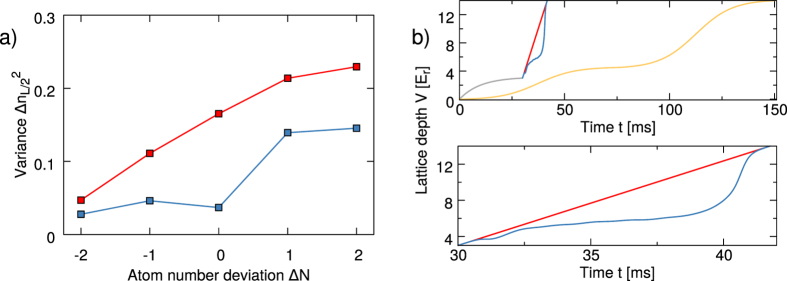
(**a**) Theoretical prediction for the final atom number fluctuation in the center of the trap 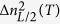 for different atom numbers *N* for the linear (red) and optimal (blue) control fields. The optimized control field works best for 16 atoms, but a small deviation only slightly decreases the figure of merit (blue). The linear ramp results in a higher figure of merit for all atom numbers (red). (**b**) Lattice ramps used in the experiment. Top: the lattice is first slowly ramped to 3 *E*_r_ (grey) before either the fast linear control field (red) or fast optimized control field (blue) is applied. The typical adiabatic control field (yellow) is much longer. Bottom: magnification of the comparison between the linear (red) and the optimal control field (blue).

Before proceeding with the experimental verification of the optimal process, we investigate its robustness with respect to the total atom number fluctuations. We show in Fig. 5a the final density fluctuations at the center of the trap 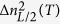 under deviations Δ*N* of the atom number up to more than 10% (i.e. Δ*N* ± 2) in the system for the optimized lattice ramp (blue). For comparison, we also show the result of a linear lattice ramp of same duration (red). As before, the optimal process displays a rather high level of robustness: the density fluctuations remain similar for all atom numbers, however larger atom numbers lead to a larger amount of defects in the density distribution. The corresponding optimal lattice ramp *V*(*t*), together with a linear ramp and the adiabatic one for reference, is displayed in Fig. 5b. The optimal protocol has been computed only for a system prepared in the ground state of the initial Hamiltonian, but we show below that the insensitivity to atom number fluctuations also provides a certain immunity against a finite initial temperature of the system.

#### Experiments

The experimental implementation of the theoretically predicted optimal protocol presented in the previous section was performed on the quantum gas microscope experiment at the Max-Planck Institut für Quantenoptik in Garching. At the beginning a two-dimensional degenerate gas of polarized Rubidium 87 atoms is produced in a single anti-node of a vertical optical lattice (period *a*_lat_ = 532 nm)^42^,^43^ (see appendix E). By slowly ramping up an additional optical lattice along the *y*-axis, the system is divided into about 10 decoupled one-dimensional tubes (both lattices had a depth of 20 *E*_r_). A third optical lattice (*x*-axis), perpendicular to the other two, is used to drive the system from the SF to the MI phase by varying its depth *V*(*t*) over time. The initial number of atoms is tuned to result in a lattice filling of one in the insulating phase, corresponding to about *N* = 16 atoms in the central tubes. At the end of this ramp, the density distribution is ‘frozen’ by rapidly raising all three lattice depths to ~80 *E*_r_. Finally, the on-site parity projected atom density is detected by fluorescence imaging^42^.

The usual adiabatic crossing of the phase transition in two dimensions to the MI phase uses a double s-shaped ramp where the slope is minimum at the phase transition. Here we use a similar ramp for the one-dimensional systems as a reference point (see yellow line in the upper panel of Fig. 5b. Each s-shaped ramp has a duration of 75 and the step is centered around the critical lattice depth of *V*_c_. This adiabatic preparation leads to an average parity 
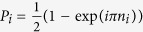
 of the site occupancy of 0.80(1) in the center of the trap. This means that 80 of the sites are filled initially with one atom, the rest being either empty or filled with two atoms (the probability for a triple occupancy can be neglected). We attribute the 20 defects mostly to the finite temperature of the initial state (we note that in a 2D geometry average final parities of >96% are typical) because this fraction does not significantly vary as we increase the duration of the lattice ramp. The theoretically predicted optimal steering field is implemented in the experimental setup by increasing the lattice depth first from 0 to *V*_i_ = 3 *E*_r_ over 30 ms and then from *V*_i_ to *V*_f_ = 14 *E*_r_ over *T*_OPT_ = 11.75 ms following the optimal control field. In order to reduce the atom number fluctuations in the experimental sample, we restrict the analysis to the central 5 tubes in two-dimensional samples having a diameter of 16 lattice sites (see appendix E).

**Figure 6:**
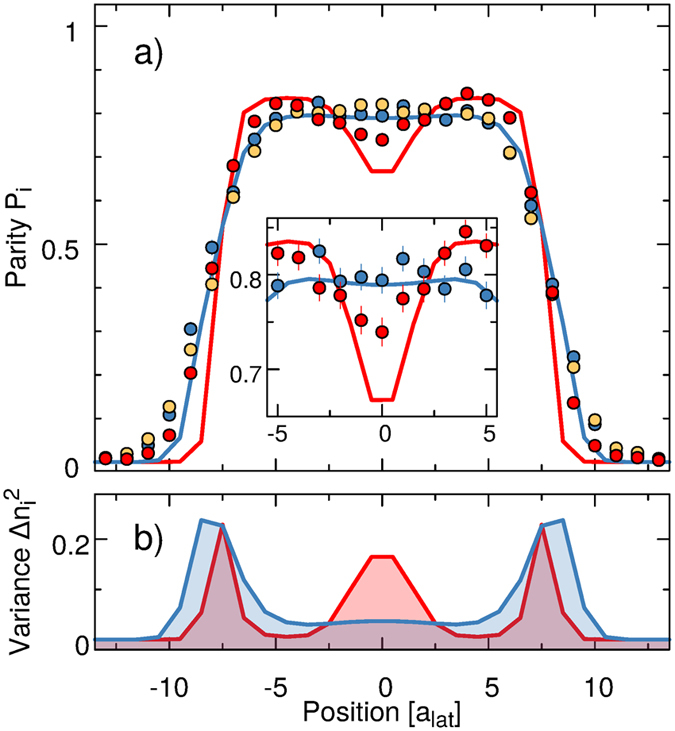
(**a**) Experimental mean parity profiles *P*_*i*_ resulting from the adiabatic (yellow points), optimized (blue points) and linear (red points) lattice ramps compared to the rescaled numerical results (blue and red lines). The short linear lattice ramp has a dip in the parity profile at the center due to non-adiabatic effects. Inset: magnification of the central part of the main panel. Standard deviations of the measured data are smaller than data points in the main plots and therefore only shown in the inset. (**b**) The red and blue shaded areas display the numerically computed atom number fluctuations for the linear and the optimized ramps lasting *T*_OPT_ = 11.75 ms.

The measured parity of the site occupancy in the final state is plotted in Fig. 6, where we compare the result of the optimal protocol to those of the adiabatic state preparation and of a linear fast ramp of the lattice performed in *T*_OPT_ = 11.75 ms. We observe no significant difference between the optimal and adiabatic protocols (yellow and blue data sets): the optimal control technique therefore leads to a factor of ten speed up of the state preparation without loss of fidelity. The performance of the optimized protocol is better appreciated when comparing with the final state reached using a linear control field of the same duration *T*_OPT_ (inset of Fig. 6). Here the parity of the site occupancy displays a dip in the center, surrounded by narrow bands of high parity. Such a distribution indicates that the redistribution of atoms across the system that is necessary to build an extended insulating region could not take place. Instead, insulating regions that formed locally around the points where the initial site occupancy was close to one have confined the excess particles in the center of the trapping potential^44^. Note that, the difference between the optimized and the linear control field — especially in the center of the trap — differs by more than three standard deviations. Moreover, in Fig. 6 we compare the experimental results with the numerical simulations for the optimal control fields: both experimental and numerical results feature a flat top profile of comparable width, but the maximum parity of the site occupancy achieved in the experiment yields *P*_max_ = 0.80(1), compared to *P*_max_ = 0.96(1) in the numerical simulation. This difference is a consequence of the simulation being performed at zero temperature, whereas the experimental system has a finite initial temperature. However, if rescaled by the experimental value of *P*_max_, there is a good agreement between them, that is, their difference can be parametrized with a single parameter corroborating the fact that their discrepancy originates most likely from the different temperature (purity) of the states.

## Discussion

We theoretically engineered and experimentally implemented two optimally controlled processes at the numerically defined quantum speed limit, for different paradigmatic complex cold atom systems: the optimal preparation of a non-classical motional state of a BEC in a magnetic trap and the 1D superfluid-to-Mott-insulator crossover of cold atoms in an optical lattice. The former experiment on cold atoms on an atom chip opens new perspectives for the development of accurate and sophisticated protocols for sensing, interferometry and cold atom manipulations. The latter results on the SF-MI crossing demonstrate that the purity of the state reached by the fast optimal protocol is the same as the one obtained by means of the adiabatic protocol, and is the first experimental demonstration of optimal control of a crossover related to a quantum phase transition in a finite system. This experiment indicates that, along the same lines, the generic adiabatic quantum computation scheme can be in principle performed in a fast and optimal way (i.e. not adiabatically).

We demonstrated that optimal processes can be engineered and implemented for many-level systems with non-linearities as well as for many-body quantum systems, and that the theoretical estimate of the QSL can be experimentally reached. We have shown numerically that the optimal processes are robust with respect to experimental imperfections, stable against atom number fluctuations (that are unavoidable without post-processing of the data) and finite temperature corrections. Moreover, the speedup of these processes naturally reduces the detrimental effects of decoherence in the system.

In conclusion, the presented results demonstrate the feasibility of optimally engineered experiments with quantum many-body systems at the QSL, and that the systematic utilization of QOC will pave the way to the experimental realization of protocols of increasing complexity in the near future.

## Methods

### CRAB optimal control

Optimal control theory is devoted to find the solution to functional minimizations of the form 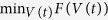, where *V*(*t*) is the control field and *F* a figure of merit to be computed via a dynamical law that describes the time evolution of the system. In QOC problems, the dynamical law is given by a Liouvillian equation for the system density matrix, which for pure states reduces to the time-dependent Schrödinger equation. Typical figures of merit are the overlap fidelity of the final state with respect to some given target state, the final energy of the system or some other interesting properties of the final state or of the path followed between the initial and the final state. Finally, figures of merit might include also constraints as the maximal power used to drive the system, the limited band-width of the control field or any other experimental constraints to be satisfied. In this work we employ the CRAB optimal control approach, that is, the optimization is implemented by looking for an optimal control field of the form *V* (*t*) = *V*_0_ (*t*) *f (t*), where *V*_0_ (*t*) is some guess function we can use to include our physical intuition on the problem, or a preexisting solution to a simplified version of the complete optimal control problem, and *f (t*) is a correction expressed in a truncated and randomized basis. For example, one could work in a truncated Fourier series of the form 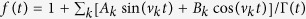 where *k* = 1,..., *n*_*f*_, *ν*_*k*_ = 2*π*(*k* + *r*_*k*_)/*T* are randomized Fourier harmonics with *T* the total time evolution, *r*_*k*_ ∈ [−1/2, 1/2] are random uniformly distributed, and Γ(*t*) is a normalization function to keep the initial and final control field values fixed. The optimization problem is then reformulated as the extremization of a multivariable function *F (A*_*k*_, *B*_*k*_, *ν*_*k*_), which can be performed with standard numerical algorithms, also gradient-free to improve their efficiency^30^. This approach has been successfully applied to different systems and protocols and it has been shown that it allows to achieve an efficient control of many-body quantum system dynamics^15^,^16^,^17^,^45^. It has also been theoretically shown that the minimal value of the final figure of merit drops exponentially with the number of optimization parameters *n*_*f*_, property that guaranties in most cases of interests an efficient and quick convergence to optimal control fields that results in errors comparable to experimental ones^18^.

### Effective one-dimensional Gross-Pitaevskii equation

The displacement of the trap, needed to excite a quasi-1D BEC as discussed in the first experiment of the paper, occurs along one of the two transverse directions, where the confinement is much stronger than in the axial direction (the frequency ratio between the transverse and the axial confinements is about 10^2^). Usually, see for instance ref. 34, a quasi-1D GPE for the axial motion is derived under the assumptions that the motion is effectively frozen to the ground state of the transverse confinement and that the atomic interactions can be described by a contact potential. Nonetheless, we shall now show that an effective GPE for the motion along one of the two transverse directions (hereafter the *y* axis) can be obtained, since the dynamics along the *y*-direction is much faster than in the axial one (hereafter the *x* axis), and therefore phononic (axial) excitations can be neglected during the transfer process.

To this end, we first compute the Heisenberg equation of motion for the atomic quantum field operator 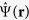. Then, we perform the replacement 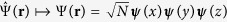 with *N* being the atom number, 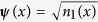, 
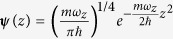
, whereas the axial atomic density of the quasi condensate is given by 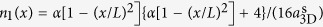 such that 
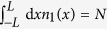
46 (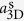 is the three dimensional s-wave scattering length). Here *m* is the atomic mass, *ω*_*z*_ the trap frequency of the harmonic confinement in the *z* direction, 
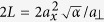
 the size of the condensate along the axial direction with 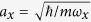, 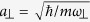, and 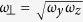. The parameter *α* is obtained by solving the algebraic equation 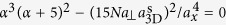. Now, by integrating over *x* and *z* the equation of motion of the matter-wave field Ψ(**r**), we obtain the effective GPE reported in Eq. (1), with 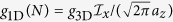, 
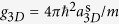
, and 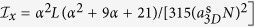. Hence, contrary to the usual coupling constant for a quasi-1D trapped atomic Bose gas^47^, in our scenario the coupling constant relies on the atom number as well. We also note that for a fixed atom number the nonlinearity in Eq. (1) is smaller than in a genuine quasi-1D quantum Bose gas, and therefore a multiorbital description of the dynamics does not provide any significant improvement to our mean field theory, as we have checked via the multi-configurational time-dependent Hartree method for bosons^26^.

Finally, we note that in order to analyze the structure of the spectrum of the optimal control field, we have solved the Bogoliubov–de Gennes equations^34^ for the GP ground (*ϕ*_0_) and first excited (*ϕ*_1_) states, that is, we solved the eigenvalue equations 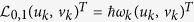, with the Bogoliubov–de Gennes operator given by^48^


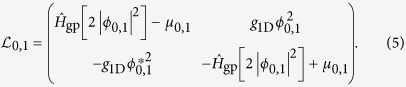


Here *μ*_0_ (*μ*_1_) is the chemical potential corresponding to *ϕ*_0_(*y*) [to *ϕ*_1_(*y*)]. For more details, we refer to ref. 48.

### BEC on atom chip experimental setup

The experimental setup consists in a quantum degenerate Bose gas of Rubidium 87 atoms trapped on an atom chip. The atom chip is a square multilayer structure covered in current-carrying gold wires. The central DC wire together with homogeneous external magnetic fields form a strongly confining anisotropic Ioffe-Pritchard trap of aspect ratio of ~200. Transversally, the trap is dressed by radio-frequency fields to form an effective slightly anharmonic potential^49^. As we outlined in the main text, the exact anharmonic potential has been approximated with a polynomial *V*(*y, t*) = *p*_2_[*y* − *λ*(*t*)]^2^ + *p*_4_[*y* − *λ*(*t*)]^4^ + *p*_6_[*y* − *λ*(*t*)]^6^, whose fit parameters are: 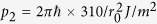, 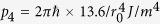, and 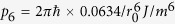 with *r*_0_ = 172 ⋅ 10^−9^ m. This experiment was performed with the potential described in ref. 31. The measured frequency in the *y* direction is *ν* = 1.77 kHz. The atomic gas is cold enough (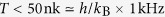) and the chemical potential small enough (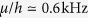 for *N*~700) that the system sits in the ground state of this potential.

To realize transfers between motional states, the potential is displaced according to the optimized control field using an external wire, located as far away from the trapping wires as possible. This simple scheme leads to a close-to-horizontal displacement, with, however, a 19° tilt with respect to the *y* direction. Excitations in the *z*-direction are anyway limited by the anisotropy of the potential but, to avoid them more completely, the angle can also be compensated for by tilting the axes of the trapping potential using the radio-frequency dressing. This configuration has been used to take the present data. However, an alternative scheme using an offset current on the radio-frequency wires has been implemented as well, enabling a purely horizontal displacement and leading to similar results.

Following the displacement in real time is not possible, but recording the evolution of the momentum distribution in the trap is. For this, the atomic cloud is released from the trap at different times during and after the transfer process, and imaged after 46 ms time of flight. These images are integrated to obtain the density distribution along the *y* direction as illustrated on Fig. 1b, and stacked to construct and represent the time evolution of the density after time-of-flight as shown on Fig. 4b. Due to the fast expansion of the cloud after the strongly confining trap is switched off, the interactions become rapidly negligible and, along the y direction, the imaged density is homothetic to the in-trap momentum distribution. The atomic cloud is imaged by fluorescence when it falls through the light sheet, which is a very thin (~40 μm) layer of laser light formed by two counter-propagating laser beams, slightly detuned from resonance. Part of the emitted photons are then captured by an objective located below the light sheet, and the atom imaged with an EMCCD camera^36^.

The efficiency of the transfer and the errorbars on it are extracted from these density evolution patterns by comparison to GPE simulations, as in ref. 31. The 1D GPE evolution of the momentum distribution in the transverse potential is calculated starting from an initial superposition of states 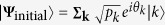 where *k* ∈ 0, 1, 2 corresponds to the ground, first excited and second excited states in the transverse direction of interest. A fit to the experimental data with the GPE simulation of the momentum distribution evolution, using the *p*_*k*_’s and *θ*_*k*_’s as fit parameters, yields the ratio of population in the first excited state, *p*_1_, which corresponds to the fidelity as defined for the optimization.

### Density matrix renormalization group

The Density Matrix Renormalization Group (DMRG) is a numerical technique tailored to one-dimensional, correlated quantum many-body systems on a lattice^24^,^25^. In its modern formulation, it exploits a tensor-network ansatz (namely a Matrix Product State) – dependent on an auxiliary dimension *χ* – to efficiently represent a many-body state with a polynomial number of free parameters as a function of the number of lattice sites. By means of different minimization techniques it is possible to obtain a faithful representation of the eigenstate properties of the system and exploiting the few-body short-range nature of the interaction among different sites and the Suzuki-Trotter formalism, it is possible to numerically simulate the time evolution of many-different setups. In particular, the process considered here can be efficiently simulated with up to tens of sites *L* and particles *N*. Recently, t-DMRG has been merged with optimal control theory by means of the CRAB optimal control technique described in appendix *A*. All simulations have been made with auxiliary dimension of up to *χ* = 24, Trotter step Δ*t* = 10^−2^ *ħ*/*E*_r_ and truncation error below *ε* ≤ 10^−5^.

### Cold atoms in the optical lattice experimental setup

For preparing a Rubidium 87 gas in a 1D geometry, we first prepare a two-dimensional (2D) gas by loading a three-dimensional BEC in a red-detuned optical lattice along the vertical *z* direction, which is the imaging direction. We then single out one of the filled lattice nodes and remove all the others by combining a strong magnetic field gradient along the lattice axis, a microwave transfer between two hyperfine states and a resonant optical pulse, as described in ref. 42. Once a single 2D gas is isolated, we adiabatically switch on (200 ms) a second optical lattice in the horizontal *y* direction, thereby forming an array of 1D tubes. The remaining tunnel coupling strength between neighboring tubes is estimated to be *J*/*h* = 5 Hz using standard band structure calculations, which has a negligible effect over the duration of the experiment. The lattice along the 1D gas (*x*-axis), which drives the transition from the superfluid to the Mott-insulating state, is turned on and slowly ramped up to 3 *E*_r_ within 30 ms. This is the initial state for the ramps to 14 *E*_r_ described in the main text.

Due to the presence of an external harmonic confinement in each direction of space, the length of the 1D gas is bound to at most 16 sites in order to maintain a filling of one atom per site in the Mott-insulating regime. For the data analysis we focused on the central 5 tubes so as to ensure that the harmonic confinement along the tubes was approximately constant. The experiment was repeated 176 times for each control field profile (linear, adiabatic and optimal), therefore yielding a statistical ensemble of 880 independent samples. Because the imaging process only gives access to the parity of the density distribution we could not sort the samples according to their atom number. We therefore included all available data in the analysis. However, based on the measured radius of the 2D gas, we are confident that the atom number in the central 5 tubes was close to 16 in average with fluctuations of the order of ±1 atom.

## Additional Information

**How to cite this article**: van Frank, S. *et al*. Optimal control of complex atomic quantum systems. *Sci. Rep.* **6**, 34187; doi: 10.1038/srep34187 (2016).

## References

[b1] Rosi G., Sorrentino F., Cacciapuoti L., Prevedelli M. & Tino G. M. Precision measurement of the Newtonian gravitational constant using cold atoms. Nature 510, 518–521 (2014). 24965653 10.1038/nature13433

[b2] Poli N. . Precision Measurement of Gravity with Cold Atoms in an Optical Lattice and Comparison with a Classical Gravimeter. Phys. Rev. Lett. 106, 038501 (2011). 21405305 10.1103/PhysRevLett.106.038501

[b3] Neumann P. . High-Precision Nanoscale Temperature Sensing Using Single Defects in Diamond. Nano Lett. 13, 2738-2742 (2013). 23721106 10.1021/nl401216y

[b4] Bloom B. J. . An optical lattice clock with accuracy and stability at the 10^−18^ level. Nature 506, 71–75 (2014). 24463513 10.1038/nature12941

[b5] Bloch I., Dalibard J. & Nascimbène S. Quantum simulations with ultracold quantum gases. Nat. Phys. 8, 267–276 (2012).

[b6] Georgescu I. M., Ashhab S. & Nori F. Quantum simulation. Rev. Mod. Phys. 86, 153–185 (2014).

[b7] Kirk D. E. Optimal Control Theory: An Introduction (Dover Pubn Inc, Mineola, NY, 2004).

[b8] Brif C., Chakrabarti R. & Rabitz H. Control of quantum phenomena: past, present and future. New J. Phys. 12, 075008 (2010).

[b9] Bhattacharyya K. Quantum decay and the Mandelstam-Tamm-energy inequality. J. Phys. A: Math. Gen. 16, 2993 (1983).

[b10] Margolus N. & Levitin L. B. The maximum speed of dynamical evolution. Physica D: Nonlinear Phenomena 120, 188–195 (1998).

[b11] Levitin L. B. & Toffoli T. Fundamental limit on the rate of quantum dynamics: The unified bound is tight. Phys. Rev. Lett. 103, 160502 (2009). 19905679 10.1103/PhysRevLett.103.160502

[b12] Deffner S. & Lutz E. Quantum Speed Limit for Non-Markovian Dynamics. Phys. Rev. Lett. 111, 010402 (2013). 23862985 10.1103/PhysRevLett.111.010402

[b13] del Campo A., Egusquiza I. L., Plenio M. B. & Huelga S. F. Quantum Speed Limits in Open System Dynamics. Phys. Rev. Lett. 110, 050403 (2013). 23414008 10.1103/PhysRevLett.110.050403

[b14] Bason M. G. . High-fidelity quantum driving. Nat. Phys. 8, 147–152 (2012).

[b15] Doria P., Calarco T. & Montangero S. Optimal Control Technique for Many-Body Quantum Dynamics. Phys. Rev. Lett. 106, 190501 (2011). 21668132 10.1103/PhysRevLett.106.190501

[b16] Rosi S. . Fast closed-loop optimal control of ultracold atoms in an optical lattice. Phys. Rev. A 88, 021601 (2013).

[b17] Caneva T., Calarco T. & Montangero S. Entanglement-storage units. New J. Phys. 14, 093041 (2012).

[b18] Lloyd S. & Montangero S. Information Theoretical Analysis of Quantum Optimal Control. Phys. Rev. Lett. 113, 010502 (2014). 25032913 10.1103/PhysRevLett.113.010502

[b19] Burgarth D. . Scalable quantum computation via local control of only two qubits. Phys. Rev. A 81, 040303 (2010).

[b20] Bücker R. . Twin-atom beams. Nat Phys 7, 608–611 (2011).

[b21] Bücker R. . Vibrational state inversion of a bose-einstein condensate: optimal control and state tomography. Journal of Physics B: Atomic, Molecular and Optical Physics 46, 104012 (2013).

[b22] D’Alessandro D. Introduction to Quantum Control and Dynamics (CRC Press, 2007).

[b23] Caneva T., Calarco T. & Montangero S. Chopped random-basis quantum optimization. Phys. Rev. A 84, 022326 (2011).

[b24] White S. R. Density matrix formulation for quantum renormalization groups. Phys. Rev. Lett. 69, 2863–2866 (1992). 10046608 10.1103/PhysRevLett.69.2863

[b25] Schollwöck U. The density-matrix renormalization group in the age of matrix product states. Ann. Phys. 326, 96–192 (2011).

[b26] Alon O. E., Streltsov A. I. & Cederbaum L. S. Multiconfigurational time-dependent Hartree method for bosons: Many-body dynamics of bosonic systems. Phys. Rev. A 77, 033613 (2008).

[b27] Cao L., Krönke S., Vendrell O. & Schmelcher P. The multi-layer multi-configuration time-dependent Hartree method for bosons: Theory, implementation, and applications. J. Chem. Phys. 139, 134103 (2013). 24116548 10.1063/1.4821350

[b28] Brouzos I. . Quantum speed limit and optimal control of many-boson dynamics. Phys. Rev. A 92, 062110 (2015).

[b29] Caneva T. . Optimal Control at the Quantum Speed Limit. Phys. Rev. Lett. 103, 240501 (2009). 20366188 10.1103/PhysRevLett.103.240501

[b30] Caneva T., Calarco T., Fazio R., Santoro G. E. & Montangero S. Speeding up critical system dynamics through optimized evolution. Phys. Rev. A 84, 012312 (2011).

[b31] van Frank S. . Interferometry with non-classical motional states of a Bose-Einstein condensate. Nat. Commun. 5 (2014). 10.1038/ncomms5009 PMC4050268 24874019

[b32] Reichel J. & Vuletic V. Atom Chips (John Wiley & Sons, 2011).

[b33] Ott H., Fortágh J. & Zimmermann C. Dynamics of a bose-einstein condensate in an anharmonic trap. Journal of Physics B: Atomic, Molecular and Optical Physics 36, 2817 (2003).

[b34] Pitaevskii L. & Stringari S. Bose-Einstein Condensation (International Series of Monographs on Physics) (Oxford University Press, USA, 2003).

[b35] Jäger G., Reich D. M., Goerz M. H., Koch C. P. & Hohenester U. Optimal quantum control of bose-einstein condensates in magnetic microtraps: Comparison of gradient-ascent-pulse-engineering and krotov optimization schemes. Phys. Rev. A 90, 033628 (2014).

[b36] Bücker R. . Single-particle-sensitive imaging of freely propagating ultracold atoms. New J. Phys. 11, 103039 (2009).

[b37] Hild S. . Far-from-equilibrium spin transport in heisenberg quantum magnets. Phys. Rev. Lett. 113, 147205 (2014). 25325657 10.1103/PhysRevLett.113.147205

[b38] Fukuhara T. . Spatially resolved detection of a spin-entanglement wave in a bose-hubbard chain. Phys. Rev. Lett. 115, 035302 (2015). 26230800 10.1103/PhysRevLett.115.035302

[b39] Brown R. C. . Two-dimensional superexchange-mediated magnetization dynamics in an optical lattice. Science 348, 540–544 (2015). 25931552 10.1126/science.aaa1385

[b40] Jaksch D. & Zoller P. The cold atom Hubbard toolbox. Ann. Phys. (NY) 315, 52–79 (2005).

[b41] Kühner T. D., White S. R. & Monien H. One-dimensional Bose-Hubbard model with nearest-neighbor interaction. Phys. Rev. B 61, 12474–12489 (2000).

[b42] Sherson J. F. . Single-atom-resolved fluorescence imaging of an atomic Mott insulator. Nature 467, 68–72 (2010). 20720540 10.1038/nature09378

[b43] Endres M. . Observation of Correlated Particle-Hole Pairs and String Order in Low-Dimensional Mott Insulators. Science 334, 200–203 (2011). 21998381 10.1126/science.1209284

[b44] Bernier J.-S., Roux G. & Kollath C. Slow quench dynamics of a one-dimensional bose gas confined to an optical lattice. Phys. Rev. Lett. 106, 200601 (2011). 21668211 10.1103/PhysRevLett.106.200601

[b45] Caneva T. . Complexity of controlling quantum many-body dynamics. Phys. Rev. A 89, 042322 (2014).

[b46] Gerbier F. Quasi-1d Bose-Einstein condensates in the dimensional crossover regime. EPL 66, 771 (2004).

[b47] Olshanii M. Atomic Scattering in the Presence of an External Confinement and a Gas of Impenetrable Bosons. Phys. Rev. Lett. 81, 938–941 (1998).

[b48] Castin Y. & Dum R. Low-temperature Bose-Einstein condensates in time-dependent traps: Beyond the $U(1)$ symmetry-breaking approach. Phys. Rev. A 57, 3008–3021 (1998).

[b49] Lesanovsky I. . Adiabatic radio-frequency potentials for the coherent manipulation of matter waves. Phys. Rev. A 73, 033619 (2006).

